# Serum neurofilament light chain measurements following nerve trauma

**DOI:** 10.1111/jns.12576

**Published:** 2023-07-05

**Authors:** Matthew Wilcox, Melissa L. D. Rayner, Owein Guillemot‐Legris, Isobel Platt, Hazel Brown, Tom Quick, James B. Phillips

**Affiliations:** ^1^ UCL School of Pharmacy University College London London UK; ^2^ UCL Centre for Nerve Engineering University College London London UK; ^3^ Peripheral Nerve Injury Research Unit Royal National Orthopaedic Hospital London UK; ^4^ UCL Medical School University College London London UK; ^5^ Institute of Orthopaedics and Musculoskeletal Science University College London London UK

**Keywords:** biomarker, diagnosis, nerve crush, nerve injury, serum

## Abstract

**Background:**

Optimal functional recovery following peripheral nerve injuries (PNIs) is dependent upon early recognition and prompt referral to specialist centres for appropriate surgical intervention. Technologies which facilitate the early detection of PNI would allow faster referral rates and encourage improvements in patient outcomes. Serum Neurofilament light chain (NfL) measurements are cheaper to perform, easier to access and interpret than many conventional methods used for nerve injury diagnosis, such as electromyography and/or magnetic resonance imaging assessments, but changes in serum NfL levels following traumatic PNI have not been investigated. This pre‐clinical study aimed to determine whether serum NfL levels can: (1) detect the presence of a nerve trauma and (2) delineate between different severities of nerve trauma.

**Methods:**

A rat sciatic nerve crush and common peroneal nerve crush were implemented as controlled animal models of nerve injury. At 1‐, 3‐, 7‐ and 21‐days post‐injury, serum samples were retrieved for analysis using the SIMOA® NfL analyser kit. Nerve samples were also retrieved for histological analysis. Static sciatic index (SSI) was measured at regular time intervals following injury.

**Results:**

Significant 45‐fold and 20‐fold increases in NfL serum levels were seen 1‐day post‐injury following sciatic and common peroneal nerve injury, respectively. This corresponded with an eightfold higher volume of axons injured in the sciatic compared to the common peroneal nerve (*p* < .001). SSI measurements post‐injury revealed greater reduction in function in the sciatic crush group compared with the common peroneal crush group.

**Conclusions:**

NfL serum measurements represent a promising method for detecting traumatic PNI and stratifying their severity. Clinical translation of these findings could provide a powerful tool to improve the surgical management of nerve‐injured patients.

## 1 INTRODUCTION

Traumatic peripheral nerve injuries (PNIs) can have devastating impacts on the quality of life of patients and represents a major global health challenge.^1^, ^2^ Patients describe deficits in the control, coordination and power of movement, reduction and alteration in appreciation of touch and sensation, and often experience chronic pain following injury.^3^, ^4^ Early detection of severe PNI and appropriate referral to specialist nerve injury clinicians is essential to ensure surgical intervention is offered where it may lead to clinical benefit.^5^, ^6^, ^7^ This is important since functional recovery following PNI is heavily influenced by the time elapsed between injury and functional muscle reinnervation. Incremental increases in the denervation time of the distal stump and end organ(s) lead to a microenvironment which becomes progressively antagonistic to nerve regeneration.^7^, ^8^ This observation is thought to be largely responsible for sub‐favourable functional outcomes seen in cases where delayed surgical intervention is deployed.^9^, ^10^ A study which measured cellular and molecular markers of human nerve degeneration reported that the tissue microenvironment may deteriorate as soon as 3‐month post‐injury.^7^ Investigations of referral times of nerve injuries requiring nerve reconstruction to specialist centres have shown that many patients are not referred until around 1‐year post‐injury.^11^, ^12^ This means the tissue microenvironment may no longer be optimal for nerve regeneration and functional reinnervation.^5^, ^7^

These data accentuate the need for improved diagnostic tools to identify PNI and facilitate prompt referral. Unfortunately, diagnostic tools used today afford a number of challenges to achieving this objective. Clinical diagnostic tools such as electromyography (EMG) and magnetic resonance imaging (MRI) analysis have emerged as the mainstay investigations to detect and monitor severe nerve injury.^13^, ^14^, ^15^ However, they are expensive to perform and difficult to interpret^15^, ^16^ without input from highly trained specialised clinicians. In many cases, it is not possible to delineate between different severities of nerve injury using these tests.^5^ Due to this issue, delays to appropriate surgical treatment are often encountered leading to worse outcomes than had earlier intervention been made.^5^, ^17^ Together, this highlights an unmet clinical need for improved diagnostic tools that are accessible in different healthcare settings, cost‐effective and straightforward to interpret.

Neurofilament light chain (NfL) is a protein that is released following axonal damage.^18^ The advent of serum NfL assays has allowed highly sensitive and specific quantification of serum NfL levels which correlate with cellular markers of neuroaxonal damage in pathologies such as traumatic brain injury (TBI) and acquired axonal neuropathies.^18^, ^19^, ^20^, ^21^, ^22^ Serum NfL assays are also significantly cheaper, less invasive and easier to interpret than EMG and MRI.^18^ Studies which link NfL assays to a volume of axonal injury could form the basis for an improved quantitative classification system of nerve injury severity that has the power to inform clinical management at an earlier stage post‐injury than is currently possible with conventional technologies.^15^, ^23^ Data which characterise: (1) the serum NfL signal seen following different severities of nerve trauma and (2) the time course of serum NfL changes after nerve injury will provide essential information that will inform the clinical utility of serum NfL in the context of nerve trauma.

Identification of appropriate human models of nerve injury through which these questions can be addressed is difficult. The broad spectrum of different trauma mechanisms means that human nerve injuries are often incomplete and mixed.^24^, ^25^, ^26^ Retrieval of human nerve tissue for study in the laboratory is often fraught with ethical and practical challenges.^26^ Low follow‐up rates among trauma patients (reported to be as low as 2%) afford difficulties to the design and deliverability of longitudinal studies.^27^

Controlled animal models of nerve injury allow researchers to retrieve tissues and monitor functional recovery at standardised endpoints for laboratory analysis.^28^ The sciatic and common peroneal nerve crush are simple and well‐controlled injuries widely used in rodents to model different volumes of axon injury.^28^, ^29^ The former represents a more proximal nerve with a larger cross‐sectional area and a longer distal segment throughout which axons will degenerate post‐injury when compared with the latter.

Using these controlled animal models of sciatic and common peroneal nerve injury, this pre‐clinical study set out to determine whether serum NfL assays can: (1) capture the presence of a sciatic or common peroneal crush and (2) accurately delineate between different severities of PNI.

## 2 MATERIALS AND METHODS

### 2.1 Sciatic and common peroneal nerve crush models in vivo

All surgical procedures were performed in accordance with the UK Animals (Scientific Procedures) Act (1986), the European Communities Council Directives (86/609/EEC) and approved by the UCL Animal Welfare and Ethics Review Board.

Forty‐six adult female Wistar rats (250–300 g) were included in the study. The rats were assigned randomly into groups housed in cages with soft bedding with free access to food and water. Each animal was deeply anaesthetised by inhalation of isoflurane, and the left sciatic nerve was exposed at mid‐thigh level. This was done by making a 3‐cm incision parallel to the femur between the knee and hip, followed by separation of the muscle layers to expose the nerve. Under the microscope (Zeiss Stemi DV4), the sciatic nerve and its branches were released from the surrounding tissue.

Crush injuries were achieved by application of consistent pressure with a pair of sterile TAAB tweezers type 4 fully closed on a specific position along the nerve (5 mm distal to the hip joint for the sciatic nerve or 5 mm from the knee joint for the common peroneal nerve; all measurements were taken with the hip in a neutral position, knee joint at 90° in flexion and the hindpaw in a neutral posture) for 15 s. This was repeated an additional two times in the same location with the tweezers positioned perpendicular to the nerve and rotated through 45° between each crush application. The injury site was marked with a 10/0 epineural suture (Ethicon).

All animals received the same level of interaction throughout the study. They were handled before surgery and throughout the study for training and completion of functional testing. All animals were given 5 min resting time before functional measurements were recorded.

After sciatic or common peroneal nerve crush, the following protocols were deployed at 1 (*n* = 5 in each group), 3 (*n* = 5 in each group), 7 (*n* = 5 in each group) and 21 days (*n* = 3 in each group) following sciatic or common peroneal nerve crush. Ten rodents did not receive any nerve injury and were used as uninjured healthy controls.

### 2.2 Serum and nerve harvest

At the endpoint of the experiment, blood samples were collected from the left ventricle using a syringe. Blood samples were transferred to BD Vacutainer tubes and left on ice for 30 min. Blood samples were then centrifuged at 2000*g* for 10 min to separate serum. Serum was then aliquoted and frozen at −80°C. The injured nerves were excised under an operating microscope. The sciatic nerve was freed and harvested 5 mm proximal to the crush site all the way down to the most distal part of the common peroneal nerve. The harvested nerve was then immediately placed in 4% paraformaldehyde (PFA) and fixed on a piece of card overnight at 4°C.

### 2.3 Nerve length measurements

Nerve samples were then removed from the 4% PFA. The following measurements of the length of nerve trunk distal to the injury site were taken using a ruler with mm divisions: (1) for the sciatic nerve injuries: the distance between the injury site and the most distal end of the harvested nerve; (2) for common peroneal nerve injuries: the distance between the injury site and the end of the common peroneal nerve. Nerve samples were then stored at 4°C in phosphate‐buffered saline until analysis.

### 2.4 Serum NfL analysis

NfL levels in serum were measured using a SIMOA® SR‐X analyser (Quanterix Corporation) with a SIMOA® NF‐light Advantage (SR‐X) Kit (Quanterix, No. 103400) according to protocols well described elsewhere.^30^ All samples were run in duplicate and operators were blinded to the experimental conditions. All samples measured were above the lower limit of detection (0.038 pg/mL) with a mean coefficient of variation of 9.15% between duplicates. Furthermore, two control samples were run on each 96‐well plate with a mean coefficient of variation of 6.90%.

### 2.5 Neurofilament staining and imaging

Cross‐sections of the sciatic and common peroneal nerve immediately proximal to the injury site were taken. Samples were incubated in formalin for 5 min, followed by 70% ethanol for 10 min, 100% ethanol for 80 min and xylene for 60 min. Samples were then embedded in paraffin wax using the Leica EG1150H embedding station. Samples were cross‐sectioned using the Leica microtome RM2235 at 5 μm and picked up on Thermo Scientific Polysine Adhesion Slides (cat no. 10219280).

Immunohistochemistry staining was performed using the Ventana Discovery XT instrument. Sections were pre‐treated using Ventana CC1 (950‐124) and incubated in 1:100 primary antibody (BioLegend anti‐Neurofilament H (NF‐H), Non‐phosphorylated Antibody SMI‐32P) for 60 min. Sections were then incubated for 60 min in 1:100 secondary antibody (rabbit anti‐mouse abcam ab98668) and the Ventana DAB Map detection Kit (760‐124) was used. Slides were then haematoxylin counterstained and a Hamamatsu S360 scanner was used to image samples.

Neurofilament‐positive area of the nerve cross‐section taken immediately proximal to the crush site was determined in nerve cross‐sections using ImageJ. This was multiplied by the length of the nerve trunk distal to the injury site to approximate the volume of the axonal degeneration in the sciatic and common peroneal nerve injury models.

### 2.6 Static sciatic index

Functional recovery was measured using the static sciatic index (SSI). The hind paw of each rat was imaged (a minimum of three images were obtained from below using a Samsung Galaxy A5 camera with the animal placed in an elevated polyethylene box) every 3–4 days following the crush injury until the endpoint of the experiment. Images were analysed using ImageJ^31^ to obtain the following parameters:

Toe Spread Factor (TSF)—the distance between the first and the fifth toes.

Intermediary Toe Spread Factor (ITSF)—the distance between the second and the fourth toes.

These values were then used to calculate the SSI using the following equation:

SSI = (108.44 × TSF) + (31.85 × ITSF) – 5.49.

TSF = TS injury – TS control.

ITSF = ITS injury – ITS control.

### 2.7 Statistical analysis

Data are presented as mean ± standard deviation (SD) unless otherwise stated. A Shapiro–Wilk normality test was conducted where appropriate. A mixed‐effects analysis was used to compare the different injury models in terms of serum NfL and SSI changes over time. In addition, ANOVA was used to compare serum NfL in the injury groups to baseline uninjured control animals. An unpaired *t* test was used to compare the distal axon volume in the two models. In all cases, *p* value of <0.05 was determined to be statistically significant. The study was powered to show twofold higher serum NfL concentration in the sciatic nerve injury group compared with the common peroneal nerve injury cohort with 90% power and an overall type I error rate of 0.05.

## 3 RESULTS

**FIGURE 1:**
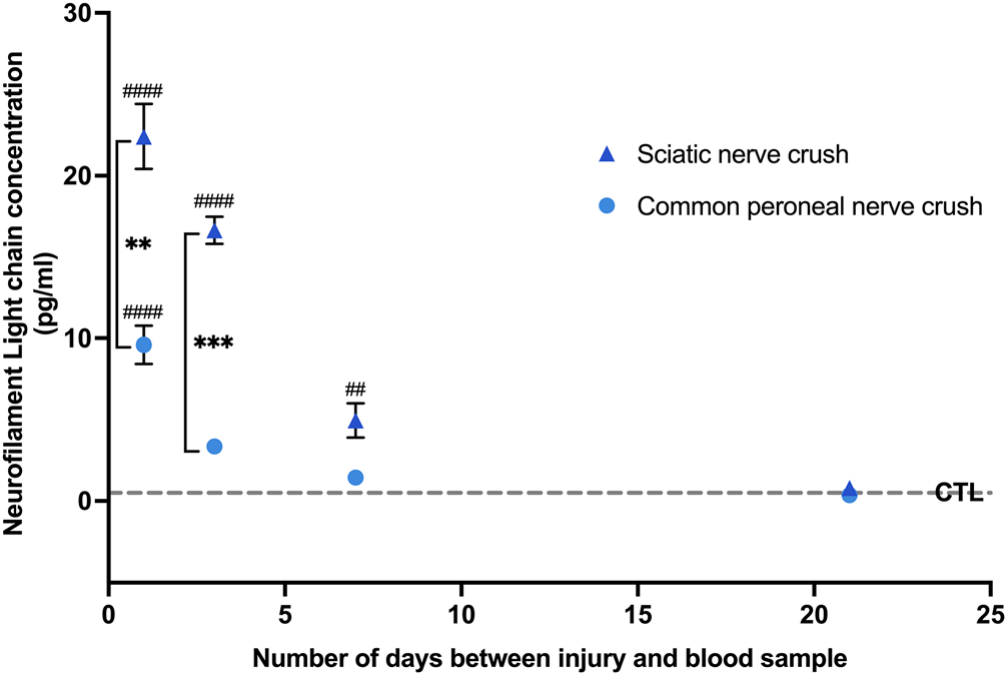
Serum concentration of neurofilament light chain (NfL) increases following nerve injury. NfL concentration was measured in serum samples retrieved from rats following surgical crush injury of the sciatic or common peroneal nerve (*n* = 5 animals for all data points except 21 days where *n* = 3). Data points represent the mean ± standard deviation. Dotted line indicates serum NfL concentration (0.05 ± 0.2 pg/mL) in uninjured control animals (*n* = 10). ***p < .001, **p < .001, mixed‐effects analysis with Sidak's multiple comparisons test comparing the two injury groups at each time point. ####*p* < .0001, ##*p* < .01, ANOVA with Tukey's multiple comparisons test comparing each injury group at each time point to baseline uninjured control.

The mean serum NfL concentration seen in healthy uninjured controls was detectable at a baseline level of 0.50 ± 0.2 pg/mL (Table 1). Serum NfL measurements were significantly higher than baseline measurements at 1‐, 3‐ and 7‐days post‐injury in the sciatic nerve crush group and at 1‐day post‐injury in the common peroneal nerve crush group (Figure 1 and Table 1). By 21‐days post‐injury, serum NfL concentration had returned to baseline levels in both injury groups. The highest serum NfL levels in both the sciatic and common peroneal nerve injury models were recorded 1‐day post‐injury; mean serum NfL levels were 45‐fold higher than baseline for sciatic nerve injuries and 20‐fold higher than baseline in the common peroneal injury group (Figure 1 and Table 1).

**TABLE 1:** Serum NfL measurements from uninjured controls and following nerve crush injury.

Number of days after injury	Nerve crush location	Mean serum NfL concentration ± standard deviation (pg/mL)
Uninjured controls (*n* = 10)	Uninjured controls	0.51 ± 0.21
1 (*n* = 5)	Sciatic	22.40 ± 3.99
1 (*n* = 5)	Common peroneal	9.6 ± 2.33
3 (*n* = 5)	Sciatic	16.64 ± 1.67
3 (*n* = 5)	Common peroneal	3.36 ± 0.25
7 (*n* = 5)	Sciatic	4.97 ± 2.12
7 (*n* = 5)	Common peroneal	1.44 ± 0.61
21 (*n* = 3)	Sciatic	0.80 ± 0.46
21 (*n* = 3)	Common peroneal	0.36 ± 0.06

*Note*: Data are displayed as a mean ± one standard deviation.

Abbreviation: NfL, neurofilament light chain.

In the sciatic nerve injury group, serum NfL readings were significantly higher compared with the common peroneal nerve injury group at 1‐ (*p* = .0046) and 3‐days (*p =* .0003) post‐injury (Sidak's multiple comparisons test). At later time points, there was no significant difference between serum NfL measurements in the different injury groups.

**FIGURE 2:**
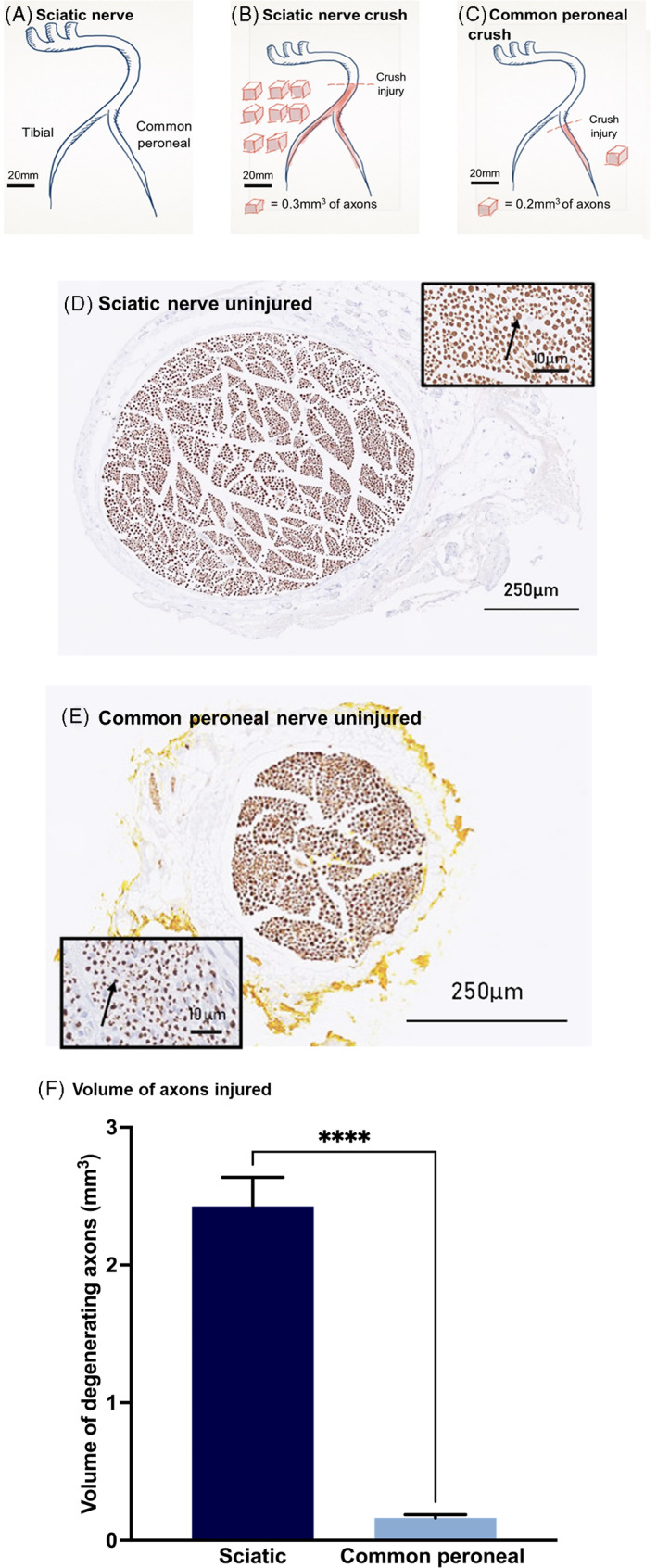
Nerve injury models and volume of axons affected. Dashed lines represent the site of the crush. One red cube represents 0.3 mm^3^ volume of axons in (B) and 0.2 mm^3^ in (C) (calculated by measuring the total neurofilament positive area in a cross‐section of the stump immediately proximal to the crush site, multiplied by the length of the distal segment). Nerve segments shaded in red represent the length distal to the crush site that were measured to determine volume of axons that degenerated distal to the injury site. (A) Intact sciatic nerve with tibial and common peroneal branches shown. (B) Sciatic nerve crush (injury performed 5 mm distal to hip joint). (C) Common peroneal nerve crush (injury performed 5 mm distal to knee joint). The black arrows in the micrographs indicate neurofilament positive staining in (D) sciatic nerve cross‐section immediately proximal to the injury site and (E) common peroneal nerve cross‐section immediately proximal to the injury site. Volume of axons injured (F) was quantified using all of the injured sciatic nerves (*n* = 18) and injured common peroneal nerves (*n* = 18), ****p < .0001, Mann–Whitney test.

To investigate the relationship between the concentration of NfL detected in the serum and the extent of nerve damage, the volume of axonal degeneration in each of the two different injury scenarios was estimated. The volume of degenerating axonal tissue in the sciatic nerve group was 15‐fold greater (*p* < .001) than in the common peroneal group (2.43 ± 0.89 mm^3^ compared with 0.16 ± 0.10 mm^3^ as shown in Figure 2D–F).

**FIGURE 3:**
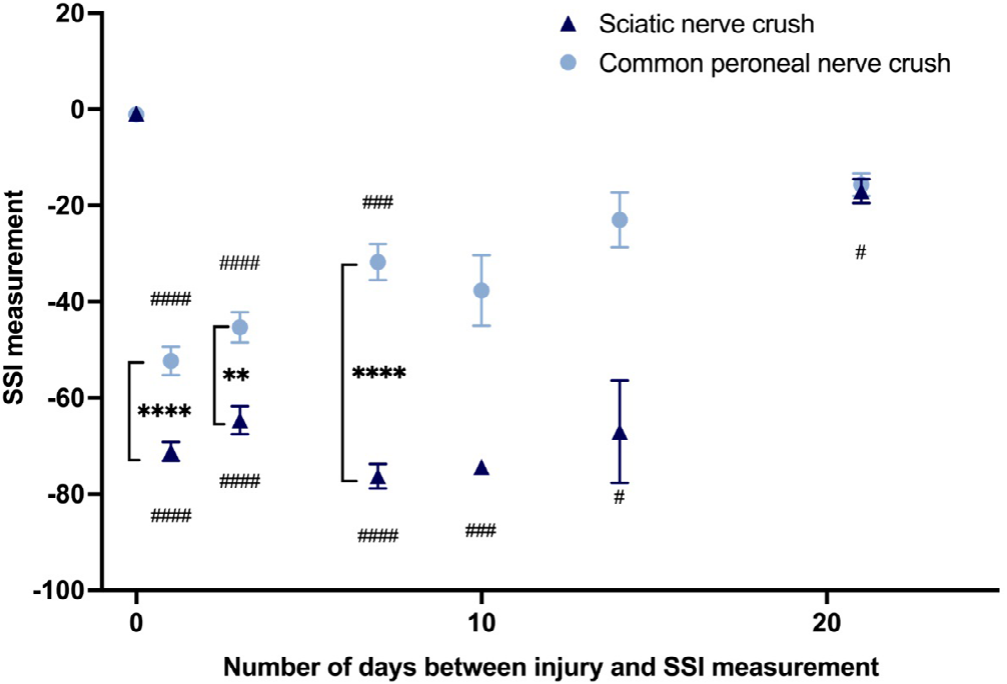
Static sciatic index (SSI) following rat sciatic and common peroneal nerve crush. SSI measurements were obtained at baseline then at various times following nerve crush. Mixed‐effects analysis indicated significant (*p* < .0001) effects of time, type of crush and interaction between the two factors. # compares each time point to Day 0 baseline in the same group, using Dunnett's post hoc test (# *p* < .05, ###*p* < .001, ####*p* < .0001). * compares sciatic to CP at each time point, using Sidak's multiple comparisons test (**p < .01, ****p < .0001).

SSI measurements revealed significantly greater functional impairment in the sciatic nerve injury group compared with the common peroneal injury group (Figure 3). Not only was the functional deficit greater in the sciatic nerve crush group, but the return to baseline was also later, with the common peroneal crush group exhibiting SSI values that were no longer significantly different to baseline after 10 days of recovery, whereas the sciatic nerve group SSI values were still significantly different to baseline at the end of the experiment (*p* = .047 at Day 21, Dunnett's multiple comparisons test).

## 4 DISCUSSION

For the first time, this study set out to obtain pre‐clinical proof of concept data for serum NfL measurements as a biomarker of nerve injury. In healthy controls, serum NfL levels were around 0.5 pg/mL, which concurs with a number of studies which have reported serum NfL readings in healthy controls.^18^, ^19^, ^20^, ^21^, ^22^

This study demonstrates serum NfL measurements can distinguish between different severities of traumatic PNI, with severity in this case being defined by the volume of axonal material distal to the crush site that would degenerate following axotomy (sciatic nerve crush yielding a 15‐fold greater volume of axonal damage and thus being classified as more severe than the smaller and shorter distal segment in the common peroneal nerve crush model). At 1‐ and 3‐day post‐injury, respectively, serum NfL readings taken from the sciatic injury group were approximately twofold to fivefold higher than those seen in the common peroneal group. This finding strengthens claims from animal models of other types of nervous system injury, such as TBI and peripheral neuropathy, which suggest serum NfL is a sensitive marker of neuroaxonal injury.^20^, ^30^, ^32^ These studies have shown that serum NfL readings are able to delineate between different cellular and molecular phenotypes of axonal degeneration. In pre‐clinical models of TBI, the level of NfL in serum peaks around 20 pg/mL compared with 8 pg/mL in peripheral neuropathy; similar to the peaks of approximately 20 and 10 pg/mL seen in the sciatic and common peroneal models of nerve injury in the present study.^20^, ^30^, ^32^

Serum NfL readings within the nerve injury groups changed over a shorter time frame and with a greater magnitude in the present study than in other pre‐clinical models of nervous system damage such as TBI and acquired axonopathies.^20^, ^30^, ^32^ The more dynamic changes seen in serum NfL readings following traumatic PNI compared to other injury mechanisms may reflect differences in biological responses to these differing injury models. A rapid cascade of biological events occur following nerve injury, which ultimately leads to the degeneration of axons distal to the injury site in the following days and weeks.^33^, ^34^, ^35^ Similarly, serum NfL readings in the acute post‐injury phase (Days 1 and 3) peaked and rapidly declined back towards uninjured levels by 1‐week post‐injury. This differs from TBI, where the neurobiological response to injury peaks within 48 h following insult but then persists for several weeks post‐injury (reflected by serum NfL readings that are approximately fourfold higher than baseline which remain at this level for 2‐week post‐injury).^30^, ^32^ In peripheral neuropathies, axonal degeneration is much slower and continues as long as there is a precipitant over weeks, months and/or years; in an animal model of chemotherapy‐induced inflammatory peripheral neuropathy, serum NfL concentrations peaked at around fourfold elevation 28 days following treatment with vincristine and have been shown to remain persistently elevated (albeit to a lesser degree) over more chronic time points.^20^, ^36^

An additional factor that may contribute to the greater magnitude and speed of changes seen in serum NfL recordings following traumatic PNI (Figure 1) compared with other neurological injury mechanisms is differentials in the interface between neuronal structures and systemic circulation. Nerve crush injuries lead to instant loss of the structural integrity of the nerve‐blood barrier^28^, ^37^ meaning that proteins such as NfL are released directly into the systemic circulation following injury. By extension, NfL can be detected in the serum rapidly following injury and metabolised. Conversely, in most central nervous system (CNS) injuries, NfL concentrations are significantly higher in the cerebrospinal fluid compared with serum.^18^, ^38^, ^39^ This differential is thought to exist since a significant proportion of NfL released following CNS insult is prevented from entering the peripheral circulation by the blood–brain barrier.^18^ Similarly, in acquired axonal neuropathies, the nerve‐blood barrier often retains structural integrity.^40^, ^41^ This may explain why in some studies, serum NfL readings lag behind key cellular and molecular events associated with CNS injury and peripheral neuropathy.^18^, ^19^, ^20^, ^21^, ^22^, ^30^ Further characterisation of the temporal behaviour of serum NfL following different severities of nerve injury could contribute to the development of a specific screening tool for PNI that could complement neurological, electrophysiological and imaging approaches. Since serum NfL readings are significantly cheaper and easier to interpret than EMG/MRI tests,^21^ this could offer particular benefit in resource poor settings where access to such technology is often challenging.^42^, ^43^

Functional recovery measured using SSI was significantly worse following sciatic nerve crush injuries when compared with the common peroneal nerve injury group. SSI readings following sciatic or common peroneal nerve crush documented in other studies behave similarly to what has been reported in the present study.^29^, ^44^, ^45^, ^46^ The significantly higher serum NfL readings at 1‐ and 3‐days post‐injury in the sciatic nerve injury group when compared with the common peroneal nerve injury group suggest that these measurements may provide a valuable predictive marker of functional outcome. This adds weight to reports which have shown significant links between serum NfL measurements and functional outcome in other neurological disorders.^18^, ^21^

A limitation of this study is that the volume of axons injured in the sciatic and common peroneal crush models was only an estimate based on the dimensions of the nerve rather than a direct quantification of the degenerating axons in the distal nerve tissue. Future studies may wish to incorporate other conventional metrics of PNI such as nerve conduction studies to compare the sensitivity/specificity of these readings with serum NfL assays. Clinical translation of data presented in this paper will require serum NfL concentrations to be tested in human paradigms of nerve injury to establish the range and timing of changes associated with nerve injuries of differing severity. One way to approach that would be to use scenarios where a controlled nerve injury is conducted following an earlier traumatic injury, for the purposes of autograft or nerve transfer.^5^ Assuming sufficient time had elapsed for any serum NfL from the initial injury to have cleared, the increase in NfL associated with the controlled nerve injury procedure could be used to establish a correlation between nerve injury severity, that is, volume of degenerated axonal tissue, and serum NfL in humans. It is often possible to retrieve excess nerve tissue from the donor nerve during these surgeries which would otherwise be discarded,^7^, ^26^ providing an opportunity for histological analysis and approximations of axonal volume loss to be made in a similar manner to the present study.

In summary, this study in a rat model has shown for the first time that peripheral nerve injury results in a substantially elevated and easily detectable increase in serum NfL concentration over the first few days following injury. Furthermore, the concentration of NfL detected is related to the volume of axonal damage, providing a sensitive measure to distinguish between nerve injuries with different severities, in turn resulting in different levels of functional recovery. If it is possible to further develop this approach in a clinical study, then serum NfL readings could become a pertinent tool for the detection, stratification and prediction of functional outcome following nerve injuries in humans. This could help to inform clinical decision‐making, for example, facilitating earlier surgical intervention where appropriate; therefore, this preclinical study is a valuable step towards driving improvements in patient outcomes.^7^, ^17^, ^23^, ^47^

## AUTHOR CONTRIBUTIONS

Matthew Wilcox conceived the study, wrote the original manuscript and performed formal analysis of the data presented. Melissa L. D. Rayner performed the investigation (animal surgeries) and reviewed the manuscript. Owein Guillemot‐Legris performed formal data analysis, data curation and visualisation, assisted with investigation (animal surgeries and collected serum samples) and edited the manuscript. Isobel Platt performed formal analysis of the SSI measurements and reviewed the manuscript. Hazel Brown reviewed and edited the manuscript. Tom Quick contributed to data interpretation, reviewed and edited the manuscript. James B. Phillips contributed to conceptualisation, supervised the study, contributed to data curation, formal analysis, reviewed and edited the manuscript.

## CONFLICT OF INTEREST STATEMENT

The authors declare no conflicts of interest.

## Data Availability

Data are available from the corresponding author upon reasonable request.
